# Comparative Effect of the Active Substance of Thyme with N-Acetyl Cysteine on Hematological Parameters and Histopathological Changes of Bone Marrow and Liver in Rat Models of Acetaminophen Toxicity

**DOI:** 10.1155/2023/1714884

**Published:** 2023-04-04

**Authors:** Zahra Mokhtari, Mahdieh Raeeszadeh, Loghman Akradi

**Affiliations:** ^1^Graduate of Faculty of Veterinary Sciences, Sanandaj Branch, Islamic Azad University, Sanandaj, Iran; ^2^Department of Basic Sciences, Sanandaj Branch, Islamic Azad University, Sanandaj, Iran; ^3^Department of Pathobiology Sciences, Sanandaj Branch, Islamic Azad University, Sanandaj, Iran

## Abstract

Acetaminophen has always been at the center of attention as a non-steroidal anti-inflammatory drug, which is generally associated with the serious side effects on liver and the hematological parameters. This study aimed to compare the effect of N-acetyl cysteine (NAC) and thyme extract on rat models of acetaminophen-induced toxicity. The present experimental study was conducted on 48 Wistar rats randomized into six groups, including the control group (no treatment); the Ac group (470 mg/kg of acetaminophen); the Ac + 100Ex, Ac + 200Ex, and Ac + 400Ex groups (acetaminophen + thyme extract at doses of 100, 200, 400 mg/kg); and Ac + NA group (acetaminophen + NAC). After weighing, a blood sample was taken from heart at the end of the period. The measured parameters were hematological, liver biochemical, and oxidative stress profiles. A part of the liver tissue was also fixed for the pathological examinations. The bone marrow was aspirated to check for cellular changes as well. The lowest mean of the final weight and liver weight to body weight ratio was observed in the Ac group. Weight loss was compensated in Ac + NA and Ac + 200Ex groups (*P* = 0.035). White blood cell (WBC), red blood cell (RBC), Hemoglobin (Hgb), and Hematocrit (HCT) in Ac and Ac + 400Ex groups showed significant differences from those of the other test groups (*P* < 0.001). Aspartate transaminase (AST), alanine transaminase (ALT), and alkaline phosphatase (ALP) enzymes in Ac + 200Ex and Ac + NA groups showed a significant decrease compared to those of the other treatment groups (*P* = 0.043). Total antioxidant capacity (TAC) and glutathione peroxidase (GPx) had the lowest levels in Ac and Ac + 400Ex groups, while malondialdehyde (MDA) had the highest content. In this regard, the liver histopathological indices (necrosis, hyperemia, and hemorrhage) in the Ac + 200Ex and Ac + NA groups reached their lowest grades in the treatment groups. The mean number of erythroid and myeloid cells in the Ac group reached the lowest (17.40 ± 3.48). The microscopic appearance of the bone marrow cells was different from normocytosis in the control group to hypocytosis in the Ac and Ac + 400Ex groups. Thymol, as an effective ingredient in thyme extract at a dose of 200 mg/kg compared to NAC, had a unique effect on reducing bone marrow and liver cell-tissue changes due to the acetaminophen toxicity.

## 1. Introduction

Acetaminophen (N-acetyl-p-aminophenol, paracetamol [APAP]) is categorized as one of the effective and recommended non-steroidal anti-inflammatory drugs (NSAIDs) in controlling inflammation and fever in the field of medicine and veterinary medicine [1]. In this regard, one of the world's most common causes of drug poisoning is acetaminophen overdose, which is considered as the second cause of liver transplantation [2].

Several drugs have been evaluated as an antidote to acetaminophen, the most important of which is N-acetyl cysteine (NAC), which is a precursor for glutathione synthesis. This metabolite is converted to water-soluble mercapturic acid by binding to glutathione and is excreted through the kidneys. Acetaminophen overdose leads to an excessive production of toxic metabolites, which in turn causes depletion of available glutathione and the development of necrosis [3].

On the other hand, hepatotoxicity is associated with changes in peripheral blood presentation [4, 5]. Changes in blood abnormalities were reported in acetaminophen poisoning since 1999. Recently, a study found the change in bone marrow stem cells (BMSCs) to be effective in improving liver damage caused by acetaminophen [6].

Thyme (*Zataria multiflora* Boiss) is a plant with analgesic, anti-inflammatory, antioxidant, anti-flatulent, blood sugar lowering, antifungal, anti-spasmodic activities, as well as protective effects on organs such as liver [7, 8].

The antiviral, antimicrobial, antioxidant, and anti-inflammatory activities of this plant are attributed to the presence of rosmarinic acid [9, 10]. In addition, thyme extract has been reported to contain anticholinergic substances [7].

NAC is the acetylated form of the amino acid L-cysteine, which is widely prescribed as an antidote for acetaminophen overdose and also as a mucolytic agent in chronic obstructive pulmonary disease. Recently, anti-inflammatory and antioxidant effects have also been reported for this drug [11, 12].

Considering the parallel clinical use of acetaminophen as a widely used NSAID and thyme, and the hepatoprotective properties of thyme, unlike acetaminophen, the present study aimed to compare the effects of thyme extract and NAC in acetaminophen overdose and to investigate the performance of these compounds in the target organs of acetaminophen including liver and bone marrow in rats.

## 2. Materials and Methods

The present experimental study was conducted on 48 adult male Wistar rats weighing 200–220 g obtained from the Center for Animal Resources and Research, Pasteur Institute of Iran. The animals were kept in an animal house with a temperature of 20–22°C, 12/12 hours light/dark cycle with free access to food and water. The study procedure was approved by the ethics committee of Islamic Azad University of Sanandaj (IR.IAU.SDJ.REC.1400.037).

### 2.1. Study Design

The animals were randomly distributed in six homogenous groups (*n* = 8 in each) as follows:

Control group: receiving normal saline by oral gavage

Group Ac: receiving acetaminophen at a dose of 470 mg/kg (dissolved in normal saline) by oral gavage

Group Ac + 100Ex: receiving acetaminophen + ethanol extract of thyme at a dose of 100 mg/kg (oral gavage)

Group Ac + 200Ex: receiving acetaminophen + ethanol extract of thyme at a dose of 200 mg/kg

Group Ac + 400Ex: receiving acetaminophen + ethanol extract of thyme at a dose of 400 mg/kg

Group Ac + NA: receiving acetaminophen + NAC at a dose of 400 mg/kg [13–15]

The LD50 of acetaminophen varies from 840 mg/kg in neonatal rats to 1220 and 1580 mg/kg in adults by oral administration [16].

At the end of the 21-day period, the animals were weighed and then anesthetized with ketamine and xylazine, and blood was taken from the heart. A part of blood with anticoagulant was used to measure the hematology parameters of white blood cell (WBC), red blood cell (RBC), emoglobin (Hgb), Hematocrit (HCT), Mean Corpuscular Volume (MCV), and Mean Corpuscular Hemoglobin (MCH). The other part was used to separate serum by centrifugation at 5000 rpm for 10 minutes to measure oxidative stress (glutathione peroxidase [GPx], malondialdehyde [MDA], and total antioxidant capacity [TAC]) and liver biochemical (aspartate transaminase [AST], alanine transaminase [ALT], and alkaline phosphatase [ALP]) parameters [17].

### 2.2. Preparation of Thyme Extract

Three kilograms of thyme plant were purchased and scientifically identified by the relevant expert. The leaves of the plant were separated from the stem and placed in the shade at room temperature to dry completely. The dried leaves were completely crushed and powdered with a grinder, and then 100 g of the resulting powder was shaken in one liter of 70% ethanol for 24 hours to obtain the extract. After filtration, the extract was concentrated in a rotary evaporator under a vacuum. Concentrations of 100, 200, and 400 mg/kg of the extract were used for subsequent experiments [15]. Moreover, the components of the extract were determined using gas chromatography–mass spectrometry (GC–MS) method.

### 2.3. GC/MS Analysis of Extract

After dehydrating with anhydrous sodium sulfate, a portion of the thyme extract sample was analyzed by a gas chromatography device connected to a mass spectrometer (Agilent 7890B GC System/5977A MSD). The injection volume was 1*μ*L and the column was HP-5 ms (30m×0.25mm) ,0.25 Micron. Moreover, the temperature of the injection site was set at 280°C. The ionization energy was 70 eV. The investigated mass range was 50–550 amu. Helium carrier gas with a purity of 999.99%, a pressure of 34 psi, and a flow rate of 1 ml/min was used. The compounds were identified by comparing their mass spectra with the data of the device databases including National Institute of Standards and Technology (NIST) and Wiley, as well as comparing the reported inhibition indices and failure patterns [18].

### 2.4. Determination of MDA Content

MDA content as a lipid peroxidation index was measured by thiobarbituric acid reactive substance (TBARS) assay using spectrophotometry based on the instructions of the kit (Cat. N: ZB-MDA-96A) manufacturer [19, 20]. The procedure was as follows: To prepare the serum sample, first 20 *μ*L of the sample was poured into the test tube. 500 *μ*L of acid reagent and 125 *μ*L of precipitation reagent were added to the contents of the tube. After 5 minutes of incubation at room temperature, it was centrifuged at 1300 rpm for 3 minutes. After discarding the supernatant, 100 *μ*L of distilled water and 2 *μ*L of butylated hydroxytoluene (BHT) were added to the sedimented material in the tube. The resulting mixture was completely vortexed and made up to 200 *μ*L with distilled water. Next, 200 *μ*L of the prepared sample was mixed with 800 *μ*L of the working solution and then placed in a Bain-Marie at 95°C for 45 minutes. In the next step, put the samples in a container containing ice water for 10 minutes to cool down quickly. Then the samples were centrifuged at 3000 rpm for 15 minutes. Next, 250 *μ*L of the supernatant was transferred to the wells, and the absorbance of the supernatant was read at a wavelength of 550 nm. After drawing the standard graph and according to the formula, the enzyme activity was measured [21, 22].

### 2.5. Measurement of Hematological Parameters

In order to perform a complete cell count test, the blood sample taken in the Complete blood count (CBC) vial containing Ethylenediaminetetraacetic acid (EDTA) anticoagulant was placed on the hematology roller mixer for 30 minutes to mix and homogenize the samples. Then the samples were given to the sysmax hematology cell counter machine. Blood cells, hemoglobin level, MCV, and MCH were measured [23].

### 2.6. Determination of GPx Activity Level

The activity level of GPx was measured by Zelbio-kit and spectrophotometry. According to the kit (ZB-GPx-48A) instructions, 50 *μ*L of the prepared sample or standard was poured into the wells of the plate. Then, 40 *μ*L of prepared R1 solution was added to all wells and incubated for 15 minutes at room temperature. To start the reaction, 10 *μ*L of R2 solution was added to the wells, and after mixing, the optical absorbance of the samples and standards was read at a wavelength of 340 nm and recorded as optical absorbance at zero time. Then the samples were incubated for 5–10 minutes at room temperature, and for the second time, the optical absorption of the samples was recorded under the title of optical absorption in the second time. After drawing the standard diagram and according to the enzyme activity formula, it was obtained [24].

### 2.7. Measurement of Activity Levels of Serum Liver Enzymes

A part of the blood (without anticoagulant) was symmetrically placed in a centrifuge at 3000 rpm for 20 minutes. After separating the serum, the activity levels of serum aminotransferases including ALT, AST, and ALP were measured by a spectrophotometric method using Pars Azmoon Kits (Pars Azmoon Co., Iran) [25].

### 2.8. Measurement of TAC

Plasma total antioxidant capacity (TAC) was measured by ferric reducing/antioxidant power (FRAP) assay introduced by Benzie and Strain (Benzie and Strain 1996) [26]. This method is based on the ability of serum to reduce ferric (Fe^3+^) to ferrous (Fe^2+^) ions in the presence of the reagent 2,4,6-tripyridyl-S-triazine (TPTZ). Hence, the end product (Fe^2+^–TPTZ) has blue color with absorption maximum at 593 nm, and the change in absorbance is related to the antioxidant capacity of the plasma [27].

### 2.9. Histopathological Examinations

A part of liver tissue was fixed in 10% formalin buffer solution. After 48 hours, the formalin solution was renewed, and the samples were kept at room temperature until the microscopic sections were prepared. After preparing tissue sections, hematoxylin and eosin (H&E) staining was done for histopathological studies. Afterwards, a semiquantitative scoring system was used to evaluate the histopathological criteria. Accordingly, at least 10 fields of each liver section were examined using a light microscope by two pathologists who were blinded to the study (Nikon E100). Liver pathology was AILI (acetaminophen-induced liver injury) scored as described previously by Muhammad-Azam et al. [28], as follows: Score 0  =  normal, score 1 (minimal–mild) = focal hepatocyte damage in less than 25% of the tissue, score 2 (mild–moderate) = focal hepatocyte damage in 25–50% of the tissue, score 3 (moderate–severe) = extensive, but focal, hepatocyte lesions in 50–75%, and score 4 (severe) = extensive, but focal, hepatocyte lesions in >75%. The morphology of observed lesions was classified and registered [29].

### 2.10. Bone Marrow Sampling

First, the femur was cut, and then the contents were removed by aspiration of sterile Phosphate buffered saline (PBS) solution inside the bone marrow. Next, one or two drops of diluted bone marrow contents were poured on a clean slide to prepare a smear. Cells were counted by trypan blue method and hemocytometer. The magnification was set at 400× [30].

### 2.11. Statistical Analysis of Data

The collected data were analyzed by SPSS23 software using the ANOVA and Duncan's post hoc test at a significance level of *P* < 0.05.

## 3. Results

### 3.1. Changes in Final Body Weight, and Liver Weight to Body Weight Ratio

Table 1 shows the initial weight, final weight, and weight gain in liver weight to body weight ratio. The highest mean weight at the end of the period was seen in the control group and the lowest in the acetaminophen group. The difference in the weight of the control group was significant compared with that of other treatment groups, except for Ac + NA. The lowest weight gain was observed in the Ac and Ac + 400Ex groups. So, this weight loss was compensated in the NAC-treated group and was not significantly different from that of the control group. The lowest liver weight to body weight ratio was seen in the Ac group and the highest in the control and the Ac + NA groups. These differences from other experimental groups were significant (*P* < 0.05).

### 3.2. Changes in the Hematological Parameters

According to Table 2, the lowest WBC count was obtained in the Ac group and the highest in the Ac + 200Ex and Ac + NA groups. A statistically significant difference was seen between the control and Ac groups with the other groups. The highest RBC count was observed in the Ac + 200Ex group, which, except for the Ac + 400Ex group, had a statistically significant difference with the other groups (*P* < 0.031). The highest mean Hgb count was obtained in the Ac + NA group and the lowest in the Ac and Ac + 100Ex groups (*P* < 0.005).

The HCT level in animals poisoned with acetaminophen in the Ac + 200Ex and Ac + NA groups was significantly increased compared to the control and Ac groups (*P* < 0.05). The results also indicated that the Ac + 100Ex and Ac + 400Ex groups had no significant difference in HCT level compared to the Ac group alone (*p* = 0.920). The highest levels of MCV and Mean corpuscular hemoglobin concentration (MCHC) were seen in the Ac + 200Ex group and then in the Ac + NA group. The lowest levels of these two parameters were also in the Ac group, which had a significant statistical difference with other groups (*P* ≤ 0.001) (Table 2).

### 3.3. Oxidative Stress Parameters

**Figure 1:**
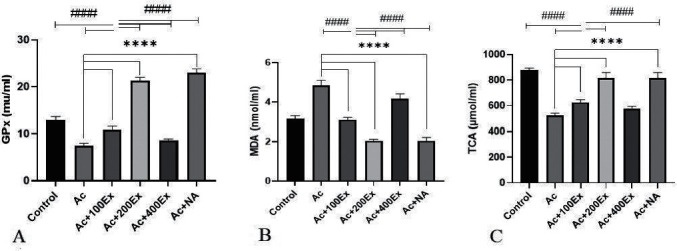
Changes in serum oxidative stress parameters in different groups. ∗∗∗∗Difference between control and Ac groups with other groups (*P* < 0.001). ^####^Difference between treatment groups with each other (*P* ≤ 0.001).

Figure 1 shows changes in the oxidative stress parameters. The highest level of GPx activity was obtained in the Ac + NA group (23.00 ± 0.88) and the lowest in the Ac group (7.53 ± 0.50). These differences were significant between the groups, except for the Ac + 200Ex group. The highest MDA content was calculated in the Ac group (4.85 ± 0.28) and the lowest in the Ac + 200Ex group (2.04 ± 0.08) and the Ac + NA group (0.18 ± 2.05). The highest level of TAC was in the control group (876.83 ± 18.45), and the lowest in the Ac group (523.66 ± 19.00). The difference in values between the control group and other groups, except for the Ac + 200Ex and Ac + NA groups, was significant at the *P* < 0.001 level.

### 3.4. Changes in Liver Marker Enzymes

**Figure 2:**
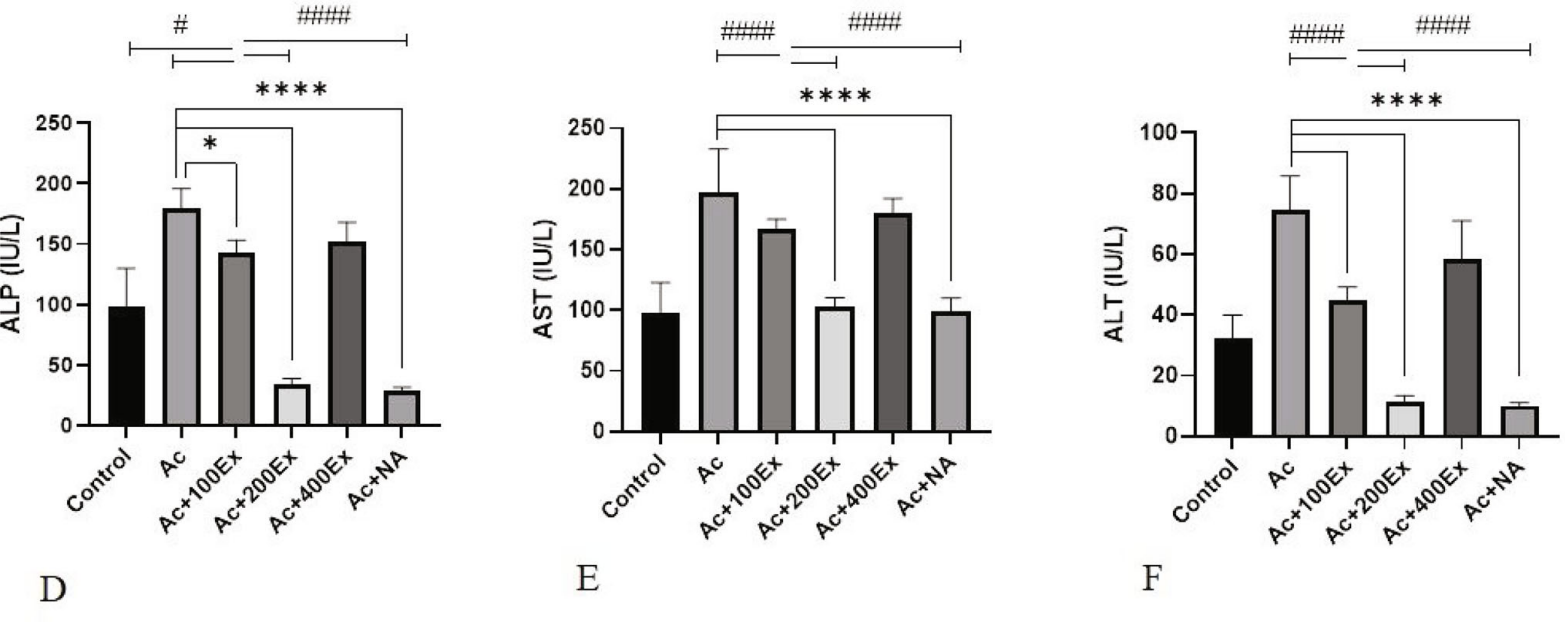
Different changes of liver enzymes in the studied groups. ^****^Difference between control and Ac groups with other groups (*P* < 0.001). ^####^Difference between treatment groups with each other *P* ≤ 0.001. ^#^Difference between control group with other groups (*P* < 0.05).

The highest level of ALP activity was in the Ac group (179.83 ± 17.40). In the treatment groups, the level of this enzyme gradually decreased, so that it reached its lowest level (29.33 ± 2.58) in the Ac + NA group, and the difference between the groups was significant. The highest level of serum AST was observed in the Ac group (196.50 ± 39.84) and in the Ac + 400Ex group (180.16 ± 13.18), respectively. The activity level of this enzyme reached its lowest level in the Ac + NA (99.33 ± 11.70), the control (97.16 ± 28.00), and Ac + 200Ex (102.66 ± 8.54) groups. According to liver damage in the acetaminophen group, liver ALT showed the highest level in the Ac group (74.66 ± 12.29) and the lowest level in the Ac + NA group (10.00 ± 1.16). The difference between the Ac + NA group, except for Ac + 200Ex, and other groups was statistically significant (*P* < 0.001) (Figure 2).

**Figure 3:**
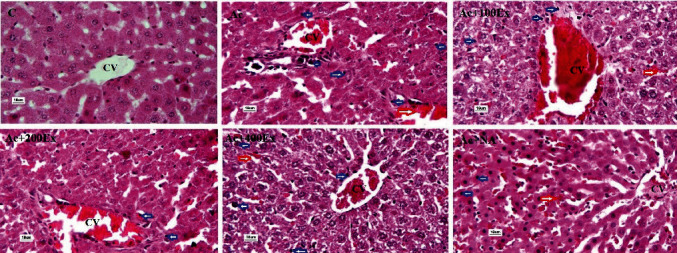
Histopathological of liver tissue sections in different study groups.Liver tissue sections in different studied groups; CV: central vein; blue arrows: hepatocyte necrosis; red arrow: hemorrhage; H&E staining, ×400 magnification.

The examined pathological lesions such as dilation and congestion in central vein, hemorrhage, and necrose are described qualitatively in Figure 3. The amount of hemorrhage and necrose between groups shows this difference. To quantify these lesions, 10 microscopic fields were examined.

According to the pathological lesion revealed in Table 3, necrose in the Ac group was significantly different from those of other experimental groups except for the Ac + 400Ex group. The treatment in the 200 mg/kg extract and NAC group was able to significantly reduce the necrosis compared to the other groups (*P* < 0.05).

**Figure 4:**
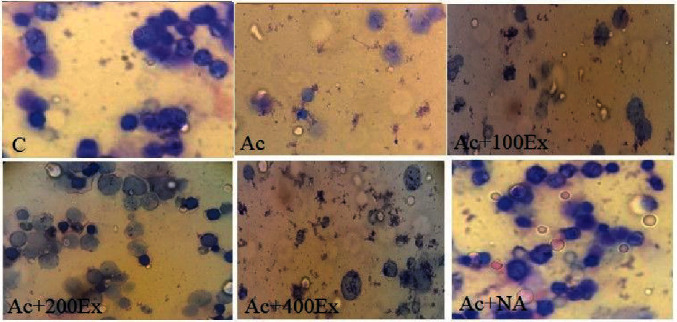
The status of bone marrow cells in different groups. Microscopic view of animal's bone marrow cells in the groups (trypan blue staining, ×400 magnification). *C* (the average number of cells is normal (normocytosis)), Ac (cell counts are reduced significantly and cellular toxicity (hypocytosis)), Ac + 100Ex (reduction of the cell counts), Ac + 200Ex (cell counts are increased and change to normocytosis), Ac + 400Ex (cell counts are decreased and cell toxicity), and Ac + NA (cell counts are increased and shift to normocytosis).

According to Figure 4, the mean number of bone marrow cells from both erythroid and myeloid lines in the Ac group has significantly decreased compared to the other groups (*P* = 0.012). The mean number of cells in the bone marrow was 38.50 ± 6.56 in the control group, 17.40 ± 3.48 in the Ac group, 24.73 ± 1.32 in the Ac + 100Ex group, 44.22 ± 4.18 in the Ac + 200Ex group, 23.38 ± 5.14 in the Ac + 400Ex group, and 47.37 ± 3.19 in the Ac + NA group.

**Figure 5:**
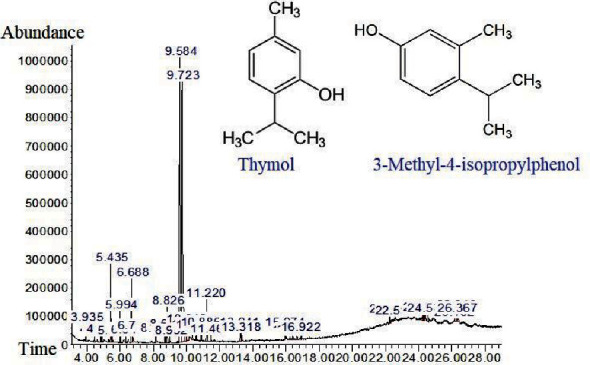
Effective ingredients of ethanolic extract of thyme.

Based on Figure 5, thymol and 3-methyl-4-isopropylphenol showed the highest concentration in the ethanol extract of thyme.

## 4. Discussion

Since acetaminophen is widely used as an antipyretic and anti-inflammatory drug in many diseases, it is important to control its side effects including liver damage and hematological changes. Therefore, the aim of this study was to investigate the effects of thyme extract compared to NAC on the liver and hematological parameters in case of acetaminophen overdose. In a recent study, an increase in body weight and liver-to-body weight ratio was observed in the groups in which the extract and N-acetyl with acetaminophen were administered. Hence, a significant reduction was found in the high dose of thyme.

Previous studies showed that the administration of ethanol thyme extract in subacute studies causes an increase in rat liver weight [31]. This is in contrast with the fact that the LD_50_ dose for thyme in rats is 980 mg/kg, and also in a 28-day study with a dose of 500 mg/kg, lung poisoning and weight loss in male rats were reported. It can be pointed out that the dose of 400 mg/kg is not safe [15, 32]. Moreover, a decrease in food intake during polyphenol poisoning has been reported in rats in the past research studies. Therefore, according to the thymol and polyphenols reported in the extract, the weight loss of animals in the condition of thyme poisoning can be pointed out [33].

The reduction of hematological parameters including WBC, RBC, HCT, and Hgb in acetaminophen overdose was reported as part of the results of this research. However, thyme extract at a dose of 200 mg/kg along with NAC was able to control this reduction and improves the status of hematological parameters.

In line with the results, Al-Awaida and Akash emphasized the protective effects of thyme extract against oxidative stress on glucose 6-phosphate dehydrogenase activity in RBCs, preventing RBC hemolysis [34]. Therefore, the reduction of oxidative stress with the antioxidant effects of the extract and N-acetylcysteine, which is one of the causes of improvement of hematological parameters in acetaminophen poisoning. The decrease in WBC count during the acetaminophen poisoning indicates the function of this drug and other NSAIDs in inhibiting the production of prostaglandins with the aid help of cyclooxygenase, resulting in the inhibition of lymphocyte proliferation [35].

In addition, paracetamol is metabolized to a toxic metabolite that damages hepatocytes and releases danger signals, thereby stimulating an innate immune response and therefore inflammation, liver tissue damage, and leukocyte depletion [36, 37].

On the other hand, considering the main target organ of acetaminophen, the most damage was in the liver tissue by inducing necrosis, bleeding, and hyperemia. Hepatic lesions were controlled in NAC and extract treatment groups. The pathological effects of liver damage were accompanied by changes in AST and ALT enzymes, the highest levels of which were seen in the acetaminophen group, and the lowest levels were seen in the NAC group, and then the dose of 200 mg/kg of thyme extract.

The increase in the serum levels of AST and ALT, which are among the important liver enzymes, during liver damage is an important diagnostic marker [38, 39].

Furthermore, Liu et al. reported 16 phenolic compounds extracted from plants and their toxic effects on rat hepatocyte cell line due to reduction of cellular energy level and death; this effect is different under vivo conditions [40]. Therefore, thymol can be declared as an effective plant substance and an anticancer compound for hepatocytic cells.

A significant increase in serum TAC, including GPx, and a decrease in MDA content indicated the antioxidant effect of thyme and NAC in acetaminophen-induced oxidative stress.

By reducing the Glutathione S-transferase (GSHt)/ oxidized gluatathione (GSSG) ratio and increasing the MDA content, acetaminophen overdose causes oxidative stress, mitochondrial dysfunction, and liver cell death (necrosis/apoptosis) [41, 42]. CYP450 system and glutathione level of the body have been proven to play a fundamental role in liver damage caused by acetaminophen overdose. Acetaminophen-induced hepatotoxicity occurs due to the activation of the CYP450 system and the production of active metabolite N-acetyl-p-benzoquinone imine (NAPQI) and its binding to GPx. Then, it turns into mercapturic acid and is excreted through the kidneys. Under the conditions of acetaminophen poisoning, when this level is higher than the capacity of glutathione, it causes liver poisoning and necrosis with covalent bonds to hepatocytes [2]. Furthermore, other studies have attributed widespread oxidative stress in acetaminophen hepatotoxicity to reactive oxygen species (ROS) produced by cytochrome P450 during APAP metabolism, which leads to an extensive lipid peroxidation and subsequent liver damage. Thereby, natural compounds, including thyme with phenolic compounds, may play a role in scavenging free radicals such as hydroxyl radicals generated by hazardous chemical agents.

Studies by Jin et al., Mazraati and Minaiyan, Azarmehr et al., and Al-Doaiss are the examples of consistent studies investigating the effects of plants containing antioxidants on the control of damage caused by acetaminophen poisoning [13, 43–45]. In this regard, the use of antioxidant compounds such as NAC and thyme was effective in reducing liver damage caused by acetaminophen-induced oxidative stress by increasing GPx concentration and improving antioxidant status.

Thyme is one of the medicinal plants with significant antioxidant properties [46]. Besides, thymol and carvacrol, as important antioxidant components of the extract, are monoterpenes whose effects are not dose-dependent [47]. Therefore, it is necessary to use and determine the effective dose in this regard [48]. In line with the results, thymol was the most active substance isolated from the extract. Due to its antioxidant effects and liver protection, this compound can be considered as the effective compound of the extract compared to the antioxidant N-acetylcysteine.

Moreover, the results of the present study also confirmed that the thyme extract was not dose-dependent so that it had a favorable effect at a dose of 200 mg/kg on the acetaminophen poisoning in the liver and bone marrow.

Soliman et al. [48] reported the hepatoprotective effects of *Thymus vulgaris*, a pungent herb of the mint family (*Lamiaceae*), at the cellular and molecular level of the antioxidant parameters GPx, SOD, and MDA, as well as the proinflammatory biomarkers Interleukin-6 (IL-6), and Tumor Necrosis Factor a (TNF a) effective in sodium nitrite poisoning. In addition, they considered the improvement of AST and ALT enzymes as a confirmation of the extract's effective performance [49].

Rašković et al. showed that the administration of thyme syrup in hepatotoxicity induced by carbon tetrachloride could significantly reduce ASL and ALT levels [50]. Regarding hemolytic anemia and the effect of acetaminophen toxicity on bone marrow, changes in erythroid and myeloid cell lines were seen in acetaminophen poisoning, so that the highest average cell count was reported in the 200 mg/kg extract and NAC groups. Changes in the average bone marrow cell count ranged from normocytic in the control group to hyptocytosis in the acetaminophen group, which was the evidence of bone marrow cell damage in acetaminophen toxicity.

Gomaa et al. [50] reported a decrease in the number of spleen cells, and an increase in bone marrow cells during the administration of acetaminophen and ibuprofen. The significant reduction of bone marrow cells found in this study can be attributed to the dose of the drugs and the animal in question. However, in regards with the dose used in the recent study, it falls under the category of toxicity studies [51].

## 5. Conclusion

As the findings of the study reveal, it could be concluded that the ethanol extract of thyme along with the antidote NAC was able to improve the liver hematological and biochemical profile as well as the pathological lesions. In addition, the mean count of erythroid and myeloid cell lines was improved in the bone marrow. Overall, the efficiency of thyme is credited to thymol and phenolic compounds of the extract for which the current study shows the cytotoxic effects at doses above 400 mg/kg. Such that the cytotoxic and anti-proliferative effects of these compounds are used to inhibit cancer cells [52]. Considering the widespread use of acetaminophen in conditions of fever and inflammation, as well as the potential of thyme due to its effective anti-inflammatory and analgesic compounds, the co-administration of these two compounds is suggested to reduce the side effects of acetaminophen on the target tissues. Examining the molecular changes in this study can be effective in completing and confirming the results.

## Figures and Tables

**Table 1:** Changes in initial weight, final weight, and liver weight to body weight ratio in the animals of different groups.

Parameters	Control	Ac	Ac + 100Ex	Ac + 200Ex	Ac + 400Ex	Ac + NA
Initial body weight (g)	205.53 ± 3.67	215 ± 6.83	213.79 ± 4.69	204.54 ± 6.79	209.59 ± 5.38	212.38 ± 5.37
Final body weight (g)	247.68 ± 5.90	220.56 ± 7.58^a,^∗	235.47 ± 4.12^a,b,^∗	236.0 ± 3.58^a,b,^∗	216.56 ± 6.17^a,b,c,^∗	243.24 ± 5.38^b,c,d,e,^∗
Weight gain (g)	42.16 ± 2.24	4.96 ± 1.34^a,^∗	21.68 ± 0.45^a,b,^∗	31.46 ± 3.48^a,b,c,^∗	6.97 ± 1.22^a,c,d,^∗	30.24 ± 1.47^b,c,d,,^∗
Liver to body weight ratio	0.36 ± 0.04	0.23 ± 0.05^a,^∗	0.26 ± 0.25^a,^∗	0.30 ± 0.03^a,b,c,^∗	0.24 ± 0.06^a,b,d,^∗	0.34 ± 0.03^b,c,d,e,^∗

Mean ± SEM of parameters in different groups. ^a,b,c,d,e^Compared to the control, Ac, Ac + 100Ex, Ac + 200Ex, Ac + 400Ex, and Ac + NA groups, respectively; ∗*P* < 0.05.

**Table 2:** Changes in hematological variables of animals in the studied groups.

	Control	Ac	Ac + 100Ex	Ac + 200Ex	Ac + 400Ex	Ac + NA
WBC (per mm^3)^	10.53 ± 0.32	6.88 ± 0.21^a,^∗	11.18 ± 0.17^a,b,^∗	13.55 ± 0.46^a,b,c,^∗	7.41 ± 0.28^a,b,c,d,^∗	13.55 ± 0.25^a,b,c,e,^∗
RBC (× 10^6^*μ*L)	8.64 ± 0.28	7.35 ± 0.37^a,^∗	7.94 ± 0.05^a,b,^∗	9.76 ± 0.36^a,b,c,^∗	7.22 ± 0.32^a,c,d,^∗	9.35 ± 0.35^a,b,c,e,^∗
HGB (g/dL)	13.01 ± 1.89	9.16 ± 0.54^a,^∗	9.19 ± 0.22^a,^∗	14.61 ± 2.58^b,c,^∗	9.75 ± 0.28^a,d,^∗	15.75 ± 1.39^a,b,c,e,^∗
HCT (%)	56.21 ± 8.27	33.31 ± 1.75^a,^∗∗∗	34.56 ± 1.46^a,^∗∗∗	59.65 ± 7.80^a,b,c,^∗∗∗	33.68 ± 1.60^a,d,^∗∗∗	58.18 ± 9.21^b,c,e,^∗∗∗
MCV (*μ*m^3^)	55.36 ± 6.51	41.75 ± 1.68^a,^∗∗∗	44.16 ± 1.34^a,^∗∗∗	61.05 ± 3.64^a,b,c,^∗∗∗	42.73 ± 2.13^a,b,d,^∗∗∗	60.15 ± 4.76^a,b,c,e,^∗∗∗
MCH (pg)	17.86 ± 1.06	12.29 ± 0.54^a,^∗∗∗	12.81 ± 0.42^a,^∗∗∗	19.22 ± 0.55^a,b,c,^∗∗∗	12.58 ± 0.34^a,d,^∗∗∗	18.98 ± 0.54^a,b,c,e,^∗∗∗

Mean ± SEM of hematological parameters in different groups. ^a,b,c,d,e^Compared to the Control, Ac, Ac + 100Ex, Ac + 200Ex, Ac + 400Ex, and Ac + NA groups, respectively; ∗*P* < 0.05, ∗∗∗*P* < 0.001.

**Table 3:** Grading of pathological liver tissue lesions in the tested groups.

Parameters	Control	Ac	Ac + 100Ex	Ac + 200Ex	Ac + 400Ex	Ac + NA
Dilation and congested central vein	0.02 ± 0.001	3.56 ± 0.48^a,^∗	1.69 ± 0.19^a,b,^∗	0.95 ± 0.24^a,b,c,^∗	0.86 ± 0.04^a,b,c,^∗	0.2 ± 0.05^b,c,d,e,^∗
Hemorrhage	0.01 ± 0.00	1.25 ± 0.48^a,^∗	0.79 ± 0.40^a,b,^∗	0.60 ± 0.04^a,b,^∗	0.90 ± 0.24^a,c,d,^∗	0.98 ± 0.43^a,b,d,e,^∗
Necrosis	0.01 ± 0.01	2.12 ± 0.38^a,^∗	0.89 ± 0.48^a,b,^∗	0.3 ± 0.05^a,b,c,^∗	2.05 ± 0.37^a,c,d,^∗	0.5 ± 0.37^a,b,c,e,^∗

Mean ± SEM of sperm concentration in different groups. ^a,b,c,d,e^Compared to the Control, Ac, Ac + 100Ex, Ac + 200Ex, Ac + 400Ex, and Ac + NA groups, respectively; ∗*P* < 0.05.

## Data Availability

The data can be obtained upon request to the corresponding author.
